# Ethics of Mitochondrial Replacement Techniques: A Habermasian Perspective

**DOI:** 10.1111/bioe.12307

**Published:** 2016-12-14

**Authors:** César Palacios‐González

**Keywords:** mitochondrial replacement techniques, mitochondrial replacement therapy, mitochondrial donation, three parent IVF, three parent babies, pronuclear transfer, maternal spindle transfer, Habermas, prenatal genetic manipulation

## Abstract

Jürgen Habermas is regarded as a central bioconservative commentator in the debate on the ethics of human prenatal genetic manipulations. While his main work on this topic, The Future of Human Nature, has been widely examined in regard to his position on prenatal genetic enhancement, his arguments regarding prenatal genetic therapeutic interventions have for the most part been overlooked. In this work I do two things. First, I present the three necessary conditions that Habermas establishes for a prenatal genetic manipulation to be regarded as morally permissible. Second, I examine if mitochondrial replacement techniques meet these necessary conditions. I investigate, specifically, the moral permissibility of employing pronuclear transfer and maternal spindle transfer. I conclude that, according to a Habermasian perspective on prenatal genetic manipulation, maternal spindle transfer (without using a preselected sperm and egg) and pronuclear transfer are morally impermissible. Maternal spindle transfer is, in principle, morally permissible, but only when we have beforehand preselected a sperm and an egg for our reproductive purpose. These findings are relevant for bioconservatives, both for those who hold a Habermasian stance and for those who hold something akin to a Habermasian stance, because they answer the question: what should bioconservatives do regarding mitochondrial replacement techniques? In fact, the answer to this question does not only normatively prescribe what bioconservatives should do in terms of their personal morality, but it also points towards what kind of legislation regulating mitochondrial replacement techniques they should aim at.

## Introduction

How should bioconservatives ethically regard mitochondrial replacement techniques (MRTs)?^1^ If they were to follow what has been advanced by bioconservative commentators, in both academic journals and media outlets, it seems that they should forcefully reject them, because MRTs are a kind of eugenic practice, go against human dignity and are germline modifications.^2^ The problem with the bioconservative assessments that have been presented up to this date is that they lack philosophical nuance; their normative conclusions seem to appear out of thin air and to be unsupported by their premises.

In order to properly understand what bioconservatives should think about MRTs, and thus to move pass the simplistic picture that they should reject all genetic biotechnological interventions directed at human prenatal life, we need to make a sophisticated and in‐depth examination of these techniques. Exploring MRTs in such a manner will have two effects. First, those who hold a bioconservative position will know, for certain, if MRTs are morally permissible for them or not, and thus whether they can resort to them or not. This, for example, is of paramount relevance for women who are bioconservatives, want to have genetically related children, want to be morally consistent, and suffer from mitochondrial DNA diseases. Additionally, bioconservatives will have a clear picture of what kind of regulation they should aim for regarding MRTs. Second, by presenting a proper account of the bioconservative position those that hold a liberal one can better challenge it. This is important if we want to avoid being self‐serving and fighting a straw man. It bears noticing that up to this date the liberal discussion on the ethics of MRTs, specifically maternal spindle transfer (MST) and pronuclear transfer (PNT), has been dominated by issues of identity,^3^ transgenerational health risks^4^, the disclosure of MRT conception,^5^ genealogical ancestry,^6^ and first in‐human use.^7^

Now, in this article I explore the morality of MRTs from a bioconservative position, specifically from a Habermasian one, thus filling an important gap in the academic literature. It is true that there is a diverse array of bioconservative positions concerning human cloning and human prenatal genetic manipulations that could be applied to the case of MRTs, for example those of Sandel,^8^ Annas^9^ or Kass.^10^ Even though this is the case I focus on Habermas's stance because his is the most sophisticated: the depth of his analysis makes *The Future of Human Nature* an unparalleled work, among the conservative ones.^11^

At this point I want to make an important methodological clarification. This work does not aim at criticising Habermas's stance on human prenatal genetic enhancing manipulations or human prenatal genetic therapeutic manipulations.^12^ I take Habermas's position regarding human prenatal genetic therapeutic manipulations at *face value* and explore how MRTs would fit into it. This entails two things. First, those who reject Habermas's stance on both enhancing and therapeutic genetic prenatal manipulations should regard this work as an exercise in Habermas's logic. Second, and most importantly, what follows from my analysis *normatively binds* bioconservatives who hold Habermas's position as true and thus they should endorse such findings, both as a matter of personal morality and in the development of regulation.

This article has three sections. In the first one I provide a scientific overview of mitochondrial diseases and present two of the methods (PNT and MST) that could be employed to allow women affected by a mitochondrial DNA (mtDNA) disease to have genetically related children free from this disease.^13^ In the second section I present and explore the three necessary conditions that Habermas establishes in *The Future of Human Nature* for prenatal genetic therapeutic interventions to be regarded as morally permissible. In the third section I explore the moral permissibility of employing PNT and MST from a Habermasian perspective.

## Mitochondrial Replacement Techniques

Mitochondria are cellular organelles that generate the energy cells need to work properly. They are characterized by: a) being *only* maternally inherited,^14^ and b) possessing their own DNA. Mitochondrial DNA comprises 0.1% of the total human DNA material; nuclear DNA comprises the other 99.9%.^15^

Mitochondrial diseases occur when mitochondria do not work properly. Problems in the mitochondria can be intrinsic to it or externally caused. *Intrinsic problems* are caused when there are deleterious mutations in the mtDNA that cause these organelles not to generate the adequate levels of energy for cells to work properly. These mutations can occur spontaneously during mtDNA replication or they can be inherited. A genetic deleterious mutation (or set of mutations) can occur across all mitochondrial genomes in a body, referred to as ‘homoplasmy’; or they can occur only in some mitochondria, known as ‘heteroplasmy’.^16^ While homoplasmic mothers will always pass the homoplasmic condition to their children, children from heteroplasmic mothers will inherit a *mix* of mutant deleterious mitochondria and healthy mitochondria. How serious a mtDNA disease will be, or if it will manifest at all, is related both to the type of mutation affecting the mtDNA and to the amount of deleterious mutant mitochondria. Additionally, *externally caused problems* can occur when genes in the cell's *nuclear* DNA affect mitochondrial function.^17^

Mitochondrial *DNA* diseases are not *one* disease, but a group of neuromuscular diseases that range in their effects from mild to devastating. Heart and major organ failure, dementia, stroke, blindness, deafness, infant encephalopathy, Leber's hereditary optic neuropathy, and premature death can all be caused by mtDNA diseases.^18^ According to the UK Department of Health, one in every 6,500 children in the UK is born with a mtDNA disease.^19^

At this time there is no *cure* for mtDNA diseases. The ‘standard’ treatment is to address the symptoms so as to increase the patient's wellbeing. This means that women who possess mtDNA diseases, know about their condition, and want to reproduce without passing on the mtDNA disease, face a complicated reproductive decision. Those who are homoplasmic carriers know that any genetically related child they have will inherit the homoplasmic characteristic. Those who are heteroplasmic know that there is a probability that their genetically related children could possess a deleterious mutant load that, if high enough, would cause the disease to manifest. Given these scenarios, women who are carriers must choose between having genetically related children (i.e. using their eggs) and face the possibility of passing on a mtDNA disease, or they can choose other reproductive options (e.g. egg or embryo donation).^20^ A third option has recently emerged. Scientists have conceived two different methods that would allow women with mtDNA diseases to have genetically related children without deleteriously mutant mitochondria: maternal spindle transfer (MST) and pronuclear transfer (PNT). It is important to stress that these techniques *only tackle mtDNA diseases*, and thus all my further discussion is narrowed to a discussion of MRTs and *mtDNA diseases*.^21^

Maternal spindle transfer (MST): assisted reproductive techniques are used to obtain eggs from the intending mother and a donor.^22^ The eggs from the mother possess deleterious mutant mitochondria while those from the donor do not. Afterwards the chromosomes, which are found on one side of the egg in a spindle‐shaped group, of both eggs are removed. The mother's chromosomes are transferred to the now enucleated donor's egg. The donor's chromosomes are discarded along with the mother's enucleated egg. At this point the reconstructed egg has *healthy* mitochondria and can be fertilized *in vitro* and then transferred into the mother or a surrogate (if there were reasons for the mother not to carry the pregnancy). The healthy mitochondria of the reconstructed oocyte will be passed down via the maternal line to subsequent generations, thereby cutting off the transmission of mitochondrial disease (so long as the chromosomes’ transfer process does not carry enough deleterious mutant mitochondria to cause heteroplasmy to the point of clinical expression of the disease).^23^

Pronuclear transfer (PNT): two zygotes are created *in vitro*. One of them is created with the intending parents’ sperm and egg (or a donor's sperm and the mother's egg), and another with a donated egg and the father's (or donor's) sperm. After the sperm has fertilized the oocyte, and during the first hours, the nuclear material of both progenitors is enclosed in different membranes that are called the male and female ‘pronuclei’. The two pronuclei are removed from both zygotes at day one in their development, and prior to their fusion. The intending parents’ (or donor's and mother's) pronuclei are then transferred to the enucleated zygote produced with the donor's egg. The reconstructed cell is then transferred into the mother or a surrogate (the resulting child would be free from mitochondrial disease as long as the chromosomes’ transfer process does not carry enough deleterious mutant mitochondria to cause heteroplasmy to the point of clinical expression of the disease). Finally, the other pronuclei and the enucleated zygote produced with the intending mother's egg are discarded.^24^

## Habermas on Prenatal Genetic Interventions

A common topic in the human enhancement debate is whether there is a moral difference between prenatal genetic therapeutic interventions and prenatal genetic enhancing interventions. Most liberal commentators argue that there is no moral difference between them and that in principle we should accept, or reject, both as ethically sound. For example, when discussing whether the notion of disease is able to ground a moral distinction between genetic therapeutic interventions and genetic enhancement interventions Nicholas Agar claims that:

The case for allowing prospective parents access to some therapeutic goods seems very strong indeed. Though gene therapy may potentially be a more effective means of combating diabetes than daily shots of insulin, it does not seem in a different moral category. Here is where the stand against eugenics is taken. If gene therapy is medicine then it should be restricted to the treatment of disease. It may be all very well to seek to correct flaws in the execution of divine or evolutionary design, but it's a different thing altogether to shape people according to our own designs. *Liberals are united in contempt for the above reasoning* [emphasis added]. They doubt that the notion of disease is up to the moral theoretic task the therapeutic/eugenic distinction requires of it.^25^

Contrary to this position, Habermas argues that there *is* a morally relevant distinction between prenatal genetic therapeutic interventions and prenatal genetic enhancing interventions. He argues, throughout *The Future of Human Nature*, that while genetic *therapeutic* interventions are morally acceptable *under certain circumstances*, genetic enhancing interventions^26^ *are not* morally permissible:

The threshold separating negative and positive eugenics can be described in terms of a difference of *attitudes*. In the framework of clinical practice, the genetic therapist treats the living being on the basis of a justifiably assumed consensus, as if the embryo were already the second person which it will one day become. Conversely, the genetic designer assumes both an optimizing and an instrumentalizing attitude toward the embryo: the eight cell embryo's genetic composition is to be improved according to subjective preferences. What takes the place of the performative attitude toward a future person, who in its embryonic state is already *treated* as a person who can say yes or no, is in the case of positive eugenics a hybrid combination of objectivating attitudes.^27^

Thus, the first condition that must be met for prenatal genetic interventions to be regarded as morally permissible is that they are of a therapeutic kind and that they are guided by a clinical attitude.

Given that the therapist treats the embryo as a second person, it is not sufficient for the intervention to be *therapeutic* and carried out according to what Habermas calls ‘the logic of healing’ for it to be morally permissible. Why not? Because standard medical practice requires that patients provide their *informed consent* to receive therapies. This being the case, there is a substantive problem: embryos cannot provide their informed consent. Habermas solves this problem by relying on the notion of ‘justifiable assumed consensus’ which means ‘presumed informed consent’. According to him, it is morally permissible to carry out genetic therapeutic interventions if we can assume, counterfactually, that the embryo would agree to the intervention if it had the required capacities: ‘[the] clinical attitude draws its legitimizing force from the well‐founded counterfactual assumption of a possible consensus reached with another person who is capable of saying yes or no.’^28^ This *counterfactual* assumption position is mostly novel in medical ethics, since the discussion of incapacity and patient's *best interests* is what has dominated the Anglophone debate so far.^29^

The second condition that must be met for a prenatal genetic intervention to be regarded as morally permissible is that we are able, counterfactually, to presume the embryo's informed consent for it.

Even if we accept presumed informed consent as morally unproblematic in the case of life saving treatments, there is a *degree of intervention issue* that must be acknowledged. How serious should a condition be for presumed consent to be legitimate? For example, how can we know that an embryo would not, counterfactually, reject a genetic *treatment*? Could a therapist presume consent for modifying genes that are related to being below average height? Habermas answers this question by asserting that we can presume consent if the genetic intervention aims at addressing conditions that are unequivocally extreme and that would be rejected by all. He writes that ‘only in the negative case of the prevention of extreme and highly generalized evils may we have good reasons to assume that the person concerned would consent to the eugenic goal,’^30^ and ‘[i]n any case, assumed consensus can only be invoked for the goal of avoiding evils which are unquestionably extreme and likely to be rejected by all.’^31^ Habermas's response sidesteps ‘problematic*’* cases, for example genetic therapy aimed at increasing height, but it is useful for the discussion of the morality of MRTs.

Finally, the third condition that must be met for prenatal genetic interventions to be regarded as morally permissible is that we aim at *preventing* extreme evils that would likely be rejected by all.

At this point we should ask if both therapeutic genetic *germline* interventions and therapeutic genetic *somatic* interventions could be morally acceptable for Habermas. The first thing to say is that in *The Future of Human Nature* he does not explicitly address the moral differences between somatic and germline interventions:

I will not go into the more specific questions of the moral responsibility we would have to take, with respect to a possible modification of the germ line, for the far‐reaching intergenerational effects of germ line therapy (banned, as yet), or even for the secondary effects of body cell therapy (…) In the following, I will refer, without further specification, to ‘genetic interventions’ which are carried before birth.^32^

However, when he discusses the ethics of PGD he tells us that: ‘A genetic manipulation (*carried out, preferably, on somatic cells* [emphasis added]) restricted to clearly therapeutic goals can be compared to the combat against epidemics and other widespread diseases.’^33^ From this quote it is clear that Habermas *is not outright rejecting* 34 germline prenatal manipulations. He is only asserting that it would be *preferable* that the interventions are carried out on a somatic level. Given that Habermas does not outright reject germline modifications the question that we must answer is: should we regard MRTs that amount to germline prenatal manipulations as *morally* permissible?

If we suppose that MRTs are effective, and that the three previous conditions are met, then it would be morally permissible, other things being equal, to select for *females* when employing them (let's remember that mitochondria are *only* inherited via the maternal line and thus when we select for females the ‘donated mitochondria’ will be passed on to future generations, if they reproduce). It would be morally permissible because the primary aim is treating ‘someone’ with a serious condition that causes pain and suffering, whereas the ‘heredity’ effect is only an unintended side effect of the procedure.^35^

## Mitochondrial Replacement Techniques: A Habermasian Perspective

At this point we can investigate the morality of both MRTs from a Habermasian perspective.^36^ The first step is to try to situate these techniques either in the therapy or in the enhancement category. At first glance it appears that both PNT and MST fall, undoubtedly, within the therapy category. Why? Because scientists seem to be guided by a clinical attitude and both techniques have been presented as *prenatal cures* for mtDNA diseases, in addition to the fact that the use of MST and PNT is not aimed to *enhance* any children or to create any *enhanced children*. At this point we are tempted to conclude that the use of MST and PNT is motivated by what Habermas calls ‘the logic of healing’ and thus that *both* techniques pass the therapy/enhancement test. The problem with this reasoning is that in fact *only* PNT and *an instance* of MST can be guided by the logic of healing; given that the logic of healing requires to be directed at *someone in specific*. It requires this in as much as ‘healing’ is a comparative term, and we can only compare states of *existing* individuals or individuals that *have existed*. In short, *healing entails healing someone*. PNT can be guided by the logic of healing because it occurs when there is already an embryo to act upon, and thus one that can be healed.

The instance of MST which can be guided by the logic of healing is that where we have preselected a specific egg and sperm for our reproductive purpose before any procedure takes place *and* before we even consider carrying out the procedure. When we have already preselected a pair of gametes, under these circumstances, we can ‘heal’ someone at the gametic stage because the preselected egg and sperm will always create the same *specific* individual. All other cases of MST cannot be considered as guided by the logic of healing because there is *no specific individual* to be healed. On the contrary, we would *create* someone with a mtDNA deleterious mutant free reconstructed egg. This is predicated on the belief that our identity is tied to the *specific* gametes that gave rise to ourselves. Let me explain this point more clearly: when we carry out MST we have a preselected egg, let's assume, but we do not know which sperm will fertilize it. We do not know this because which sperm will fertilize the reconstructed egg is dependent on a series of highly mutable factors, for example the amount of healthy sperm found in the sample, the time of collection of the sample, etc. This means that the selection of the sperm will determine who comes into existence, and thus prior to such selection, if it takes place, no specific individual can be said to be healed.

In conclusion, PNT and MST with preselected gametes (henceforth MSTpg) pass the therapy/enhancement test, falling on the therapy side. On the other hand, MST without preselected sperm (henceforth MSTwps) falls neither on the therapy side nor on the enhancement side. It falls under the scope of negative eugenics understood as ‘*improving the* ‘*gene pool’ by reducing the prevalence of (people with) subnormal traits (health‐related or otherwise)*.’^37^ Does this mean that a Habermasian stance on prenatal genetic therapy cannot account for MSTwps? In a strict sense it cannot. Throughout *The Future of Human Nature* Habermas only deals with technologies that can be applied at the embryonic stage, and this is a theoretical limitation of Habermas's account when considered against the broader topic of genetic engineering.

One technique that comes to mind when talking about a preselected sperm and egg for our reproductive purposes is intracytoplasmic sperm injection (ICSI), which could be used after MST. In this technique a single sperm is selected and injected into the cytoplasm of an egg, which is then transferred to the intending mother or a surrogate. In order to establish if ICSI would satisfy the conditions of MSTpg what we need to examine is whether the gamete selection occurs before any procedure takes place and before we even consider carrying out the procedure. Establishing if these conditions are met is important, because if *they are not* then the decision to employ ICSI causally affects which sperm will be selected, and thus who will be brought into existence. When these conditions are not met employing ICSI, after MST, is equal to MSTwps, since we do not know the identity of the sperm that will fertilize the egg.

Now, the second step is to examine if PNT and MSTpg would prevent ‘extreme and highly generalized evils’ and therefore be considered as morally permissible. There is not a straightforward answer to this question, given that the effects of mtDNA diseases vary from mild to devastating. It should be clear that it would be morally permissible to use MSTpg and PNT to avoid the devastating versions of mtDNA diseases. However, even when PNT and MSTpg can be morally employed in order to avoid extreme evils there is a problem regarding the moral permissibility of their use in other instances. The problem is that we *are unable to accurately predict* what degree of evil, if any, an individual will suffer as a result of having a deleterious mutant load of mitochondria. This means that, for most cases, we do not know if the use of MSTpg or PNT counters the occurrence of mild, moderate, or devastating evils. We do know that homoplasmic mothers will always pass the deleterious mutant mitochondria to their genetic offspring, but we are unable to ascertain if the children will be symptomatic or asymptomatic, although there is a high probability of symptoms developing. The case of heteroplasmic mothers is even more difficult. We cannot accurately predict the gravity of the disease that children of heteroplasmic mothers would develop, if any, or the degree of deleterious mutant load that they will possess.^38^

Someone could propose the use of preimplantation genetic diagnosis (PGD) to investigate the status of the deleterious mutant load and then allow, or not, the use of PNT and MSTpg. The problem with this option is that PGD involves extracting a cell from an embryo that has passed the *one‐cell* stage, and then testing it.^39^ Resorting to PGD *necessarily implies* that we would be unable to afterwards use MSTpg or PNT, because MSTpg takes place *before* fertilization and PNT takes place at the *one‐cell stage*. Therefore, PGD would not serve as an adequate tool to indicate the moral permissibility of carrying out MSTpg or PNT. We must conclude, at this point, that, from a Habermasian perspective, it would *only* be morally permissible to use PNT or MSTpg when we know, *accurately* and *in advance*, that an extreme evil would be prevented by such techniques.^40^

A second theoretical shortcoming of Habermas's position is manifest when dealing with genetic conditions that appear within a spectrum that ranges from mild to devastating. It seems that Habermas is oblivious to the fact that *most* genetic conditions manifest in varying degrees due to other intrinsic and extrinsic factors. For example, the genes BRAC1 and BRAC2 have been associated with breast and ovarian cancer, but their presence does not mean that cancer will necessarily ensue.^41^ If we agree with Habermas's thoughts on the *severity threshold*, then we would have to accept that it would be morally *impermissible* to carry out prenatal genetic therapy to alter these genes, if this was possible. Habermas's theoretical shortcoming is that for all genetic conditions that manifest within a spectrum, we cannot morally intervene if we do not accurately and *a priori* know how great the evil will be. This means that most of the time we would be morally unable to intervene with prenatal genetic therapy, even if afterwards the condition revealed itself to be devastating.

So far it seems that in most cases MSTpg and PNT should be regarded as morally impermissible. While this is the case in my above take on Habermas's work, I think there is an alternative position that would allow for a more liberal use of PNT and MSTpg. Throughout *The Future of Human Nature* Habermas alternates between two positions for allowing prenatal genetic therapeutic interventions. The first position, to which Habermas gives substantial weight, was just presented. According to this one, prenatal genetic therapies can be considered as morally permissible *only if* they will prevent *extreme evils*.^42^ The main problem with this position, as I have explained, is that it rules out therapeutic interventions for conditions that *could* cause extreme evils, because of the uncertainty that they will actually do so. This position is incapable of dealing with the concept of ‘risk’, because Habermas's is mainly thinking of monogenetic diseases.^43^ I contend that this position is too demanding and that Habermas would agree to therapeutically alter genes associated with diseases such as, for example, cancer if the interventions were safe enough.

The second position, which I consider is more tenable but less explored within *The Future of Human Nature*, is that the threshold for accepting prenatal genetic therapeutic interventions as morally permissible is related to the fact that they would prevent *evils likely to be rejected by everybody*.^44^ If we accept this position as correct then we can conclude that it would be morally permissible to carry out MSTpg and PNT in order to avoid mtDNA diseases. It would be so because both techniques would prevent *unmistakeable* evils that are likely to be rejected by all, but more importantly that seem to be rejected by those currently affected by mtDNA diseases.

Three things should be noted at this time. First, Habermas is endorsing a bad‐difference view of disabilities, since he characterizes the effects of genetic conditions as ‘evils’.^45^ Second, this second position would reject removing the genetic causes of deafness (a paradigmatic study case within medical ethics) given that such condition is not likely to be rejected by all.^46^ Third, it is true that certain people *oppose* MST and PNT, but the point here is not whether they oppose *these techniques*, but whether they oppose the evils caused by mtDNA diseases.

The third step is to ask if we can, counterfactually, assume the embryos's consent for the prenatal genetic therapy. While I previously quoted Habermas stating that consent can only be presumed for the prevention of *extreme* evils^47^ in another place he also maintains that ‘[a]s long as medical intervention is guided by the clinical goal of healing a disease or of making provisions for a *healthy life*, the person carrying out the treatment may assume that he has the consent of the patient preventively treated.’^48^ This means that we can presume informed consent for the prevention of non‐extreme evils when our actions are guided by the clinical goal of healing or of making provisions for a healthy life. Now, given that genetic therapists are guided by the logic of healing when treating mtDNA diseases, with PNT and MSTpg, then we can, counterfactually, assume the embryos’ consent. We can do so if we accept Habermas's second position on establishing the validity of presumed consent, and reject the first one.

At this point we can conclude that, according to a Habermasian perspective on prenatal genetic therapeutic interventions, MSTpg and PNT (for selecting males and females) are morally permissible, and that MSTwps falls outside Habermas's theoretical framework as presented in *The Future of Human Nature*. It is very important to acknowledge a caveat about the above conclusion: it was reached without taking into account Habermas's discussion of the value of human prepersonal life. In order to have a complete picture of the morality of MRTs, from a Habermasian perspective, we would need to include his stance on this issue.

Contrary to some conservative positions, Habermas does not consider that the human embryo is a person from the moment of conception. According to him a human being becomes a person when she enters into the public sphere of a linguistic community: “As a member of a species, as a specimen of a community of procreation, the genetically individuated child in utero *is by no means a fully fledged person *‘*from the very beginning*’.’’^49^ [emphasis added]. Notwithstanding this assertion, Habermas does not think that human embryos can be liberally used for medical research, or included in the balancing of competing goods.^50^ Why? Because he maintains that human embryos’ human life possesses certain value; a value that does not make them inviolable but that nevertheless makes them to qualify as beings that should ‘not be disposed over’:

Both sides [of the abortion debate], it seems, fail to see that something may be ‘not for us to dispose over’ and yet not have the status of a legal person who is a subject of inalienable human rights as defined by the constitution. It does not solely belong to human dignity to qualify as ‘not to be disposed over’ [‘unverfügbar’]. Something may, for good moral reasons, be not for us to dispose over and still not be ‘inviolable’ [‘unantastbar’] in the sense of the unrestricted or absolute validity of fundamental rights (which is constitutive for ‘human dignity’ as defined in Article 1 of the Basic Law).^51^

Habermas's distinction between inviolability and ‘not to be disposed over’ (here we should understand ‘not to be disposed over’ as ‘not to use’)^52^ seems to allow him to maintain a ‘liberal’ position regarding abortion, and at the same time a conservative position regarding the destruction of human embryos for medical research. Abortion is morally permissible because it ‘just’ destroys human embryos, whereas medical research on human embryos ‘uses’ them and thus is morally impermissible. If we grant Habermas's point and accept that human embryos can be destroyed but *cannot* be ‘disposed over’ (that is, cannot be ‘used’), then we have also to accept that we should rule out PNT as morally permissible. Because PNT requires that we *dispose over* (i.e. ‘use’) the embryo produced with the donor's egg and father's (donor's) sperm. We dispose over this embryo in that we use its biological parts, while destroying it, for the sake of the intending mother's reproductive project. This being the case, we need to conclude that from a Habermasian perspective PNT is morally impermissible, because it requires that we dispose over (or ‘use’) a human embryo in order to benefit the intending mother. MSTpg, on the other hand, is immune to this issue given that is occurs at the gametic stage and thus we do not dispose over any human embryo.^53^ This means that from a Habermasian perspective on MRTs *only* MSTpg is morally permissible.

## Conclusion

In this article I investigated the ethics of MRTs from a Habermasian perspective. The first step was to classify MST and PNT either as therapy or enhancement. I concluded that PNT is a form of prenatal genetic therapy and thus it is morally permissible according to this standard. MST, on the other hand, should be divided in two: MSTpg and MSTwps. MSTpg can also be regarded as a form of prenatal genetic therapy given that the identity of the future human is fixed by virtue of the preselection of a sperm and an egg for the reproductive purpose, when the preselection is made before any procedure takes place *and* before we even consider carrying out the procedure. On the other hand, MSTwps cannot be regarded as genetic therapy given that there is no one to be healed. MSTwps is a type of creation act that falls within a certain definition of negative eugenics, not discussed by Habermas.

The second step was to examine if the kind of evils PNT and MSTpg would prevent passed Habermas's threshold of moral permissibility for prenatal genetic therapeutic interventions. Both PNT and MSTpg did not pass the threshold of moral permissibility, when moral permissibility is established solely at the point of preventing extreme evils. They did not pass it because mtDNA diseases do not always manifest in the same ways and thus we are unable to predict if MSTpg or PNT would prevent an extreme, moderate, or mild evil. But, on the other hand, both MSTpg and PNT pass the threshold when we adopt an alternative position regarding the moral permissibility of prenatal therapeutic interventions. According to the latter position, an intervention is morally permissible if it prevents unmistakeable evils likely to be rejected by all.

As a third step I investigated if we could presume consent for carrying out MSTpg and PNT. In this case there were also two positions. According to the first position we cannot presume consent for carrying out MSTpg and PNT, because it is not certain that they will prevent extreme evils. The second position, which seems more tenable, maintains that if the interventions are guided by the logic of healing and they aim at making provisions for a healthy life then we *can* presume consent. If we endorse the second position then it follows that we can in fact presume consent for MSTpg and PNT, given that they are guided by the logic of healing and they aim at benefiting the health of the future second person.

Finally, I examined Habermas's stance on the value of human embryos and how this relates to MRTs. I concluded that while MSTpg would be morally permissible PNT would not. PNT would not be morally permissible because it requires that a human embryo is disposed over, in order to benefit the intending mother's reproductive project. On the other hand, MSTpg is morally permissible because it takes place at the gametic stage and thus it does not require a human embryo to be disposed over.

At this point it must be clear that during my assessment of MRTs I considered these techniques as *finished*, by which I mean that they are already available in clinical practice and that their research and development phase has long passed. If we do not consider them as *finished*, but as techniques that are currently under development, then a further *fatal* complication must be noted: MST research requires the destruction of human embryos. MST research requires the destruction of human embryos because in order to establish if the technique is effective scientists need to perform MST and then create embryos only for the purpose of researching upon them, for example in order to investigate embryo development post‐MST. And after such research ends these embryos will be discarded. This means, at this point in time and from a Habermasian stance, that we also need to reject MSTpg: given that *current research on MST disposes over* human embryos.

Now, the above conclusions are relevant for bioconservatives who follow Habermas in two ways. The first one is that if they want to be morally consistent, as a matter of personal morality, then in the actual state of affairs they should reject *all* MRTs at this point. This conclusion is particularly interesting because *in principle* MSTpg could be regarded as morally permissible, but the conditions for this to be the case are *almost impossible to attain*. The second way in which these conclusions are relevant for such bioconservatives is that they should inform what kind of legislation they should support when developing regulations on MRTs. If bioconservatives think that public policy regarding new reproductive techniques should follow ethical analysis then, at this present moment, they should oppose and try to block any legislation that would approve the use of MRTs, such as the one recently passed in the UK. Finally, in a debate where the bioconservative positon has been presented as naïve and rabidly anti‐biotechnological, my findings show that the reasons a Habermasian account has to reject MRTs are complex and varied in nature. This means that there is no single silver bullet for taking down Habermas's position, but that we should seek to engage with each identified independent reason for rejecting MRTs.

## Footnotes

1
This name – mitochondrial replacement techniques – has generated controversy, since it is a misnomer (in actuality it is *not* mitochondria which are being transferred but nuclear material). See: S.A. Newman. *FDA Asked to Approve Creation of Genetically Modified Children* Huffington Post. 2014. Available at: http://www.huffingtonpost.com/stuart-a-newman/fda-asked-to-approve-crea_b_4809876.html [accessed 2 April 2014]; S.A. Newman. *Deceptive Labeling of a Radical Embryo Construction Technique* Huffington Post. 2014. Available at: http://www.huffingtonpost.com/stuart-a-newman/deceptive-labeling-of-a-r_b_6213320.html [Accessed 17 December 2014]; V. Ravitsky, S. Birko, R. Dupras‐Leduc. The ‘Three‐Parent Baby’: A Case Study of How Language Frames the Ethical Debate Regarding an Emerging Technology. *Am J Bioeth* 2015; 15(12): 57–60. For a justification of why I use the term ‘mitochondrial replacement techniques’ see C. Palacios‐González. Mitochondrial replacement techniques: egg donation, genealogy and eugenics. *Monash Bioeth Rev* 2016; 34: 37–51. 10.1007/s40592-016-0059-xPMC501653627468863

2
C. MacKellar. 2014. *Should persons affected by mitochondrial disorders not be brought into existence?* BioNews. Available at: http://www.bionews.org.uk/page_385343.asp [Accessed 13 May 2016]; C. MacKellar. *Questions relating to ‘mitochondrial replacement’* BioNews. Available at: http://www.bionews.org.uk/page_395064.asp [Accessed 13 May 2016]; S.A. Newman. 2013. *The British Embryo Authority and the Chamber of Eugenics* The Huffington Post. [Online]. Available at: http://www.huffingtonpost.com/stuart-a-newman/mitochondrial-replacement-ethics_b_2837818.html [Accessed 25 Jan 2016]; F. Baylis. The ethics of creating children with three genetic parents. *Reprod Biomed Online* 2013; 26(6): 531–534. 10.1016/j.rbmo.2013.03.00623608245

3
A. Wrigley, S. Wilkinson, J.B. Appleby. Mitochondrial Replacement: Ethics and Identity. *Bioethics* 2015; 29(9): 631–8. 10.1111/bioe.12187PMC462070326481204

4
J.B. Appleby. The ethical challenges of the clinical introduction of mitochondrial replacement techniques. *Med Health Care Philos* 2015; 18(4): 501–14; Baylis. *op. cit* note 2; M.H. Johnson. Tri‐parenthood–a simply misleading term or an ethically misguided approach? *Reprod Biomed Online* 2013; 26(6): 516–9; A.L. Bredenoord, W. Dondorp, G. Pennings, G. De Wert. Avoiding transgenerational risks of mitochondrial DNA disorders: a morally acceptable reason for sex selection? *Hum Reprod Oxf Engl* 2010; 25(6): 1354–60. 10.1093/humrep/deq07720364025

5
Appleby. *op. cit* note 4; J. Harris. Germline Manipulation and Our Future Worlds. *AJOB* 2015; 15(12): 30–34; J. Harris. Germline Modification and the Burden of Human Existence. *Camb Q Healthc Ethics* 2016; 25(1): 6–18; I. de Melo‐Martin I. When the Milk of Human Kindness Becomes a Luxury (and Untested) Good. A Reply to Harris’ Unconditional Embrace of Mitochondrial Replacement Techniques. *Camb Q Healthc Ethics* Forthcoming; J. Harris. How to Welcome New Technologies: Some Comments on Inmaculada De Melo‐Martin’. *Camb Q Healthc Ethics* Forthcoming; C. Palacios‐González. Resource Allocation, Treatment, Disclosure, and Mitochondrial Replacement Techniques: Some Comments on de Melo‐Martin and Harris. *Camb Q Healthc Ethics* Forthcoming.

6
Baylis. *op. cit*. note 2.

7
A.L. Bredenoord, P. Braude. Ethics of mitochondrial gene replacement: from bench to bedside. *BMJ* 2010; 341(nov08 1): c6021–c6021. 10.1136/bmj.c602121059727

8
M.J. Sandel. 2009. *The Case Against Perfection*. Cambridge, Mass: Belknap Press.

9
G.J. Annas. Why we should ban human cloning. *N Engl J Med* 1998; 339(2): 122–5. 10.1056/NEJM1998070933902139669897

10
L.R. Kass. Wisdom of Repugnance: Why We Should Ban the Cloning of Humans, *The. Valpso Univ Law Rev* 1998; 32(2): 679–705. 12710451

11
J. Habermas. 2003. *The Future of Human Nature*. Cambridge, UK: Polity Press.

12
For a recent critique of Habermas's arguments against genetic prenatal enhancing interventions see, J. Pugh. Autonomy, Natality and Freedom: A Liberal Re‐examination of Habermas in the Enhancement Debate. *Bioethics* 2015; 29(3): 145–52. 10.1111/bioe.12082PMC434702024547807

13
For a good summary of the regulatory discussions surrounding MRTs in the US and UK see, Appleby *op. cit* note 4; I.G. Cohen, J. Savulescu, E.Y. Adashi. Transatlantic lessons in regulation of mitochondrial replacement therapy. *Science* 2015; 348(6231): 178–80. 10.1126/science.aaa815325859028

14
The advent of in vitro gametogenesis could change this fact. If functional oocytes were to be generated from males then mitochondria could, in such cases, be inherited from the genetic father that provided the cell from which the egg was derived. C. Palacios‐González, J. Harris, G. Testa. Multiplex parenting: IVG and the generations to come. *J Med Ethics* 2014; 40(11): 752–8. 10.1136/medethics-2013-101810PMC421529124608087

15
R.W. Taylor, G.A. Taylor, S.E. Durham, D.M. Turnbull. The determination of complete human mitochondrial DNA sequences in single cells: implications for the study of somatic mitochondrial DNA point mutations. *Nucleic Acids Res* 2001; 29(15): e74–e74. 10.1093/nar/29.15.e74PMC5583911470889

16
Throughout the text I will use the term homoplasmic mothers to refer to those women who are homoplasmic for a deleterious mutation of the mtDNA.

17
Nuffield Council on Bioethics. 2012. *Novel techniques for the prevention of mitochondrial DNA disorders: an ethical review*. London: Nuffield Council on Bioethics: vii.

18
Department of Health (DH). 2014. *Mitochondrial Donation: A consultation on draft regulations to permit the use of new treatment techniques to prevent the transmission of a serious mitochondrial disease from mother to child* Available at: https://www.gov.uk/government/uploads/system/uploads/attachment_data/file/285251/mitochondrial_donation_consultation_document_24_02_14_Accessible_V0.4.pdf [Accessed 5 Jan 2015]

19
A.M. Schaefer, R. McFarland, E.L. Blakely, L. He, R.G. Whittaker, R.W. Taylor, et al. Prevalence of mitochondrial DNA disease in adults. *Ann Neurol* 2008; 63(1): 35–9; Department of Health (DH). *op. cit*. note 18. 10.1002/ana.2121717886296

20
It has been suggested that preimplantation genetic diagnosis (PGD) can be used for selecting embryos that do not possess a deleterious mutant mtDNA load to the point of clinical expression of the disease. The problem with this proposal is that is useless for homoplasmic women, and that it only serves heteroplasmic women, at this point, if there is enough data on the specific mtDNA mutation. PGD would not be useful for mutations that are uncommon or private. H.J. Smeets, S.C. Sallevelt, J.C. Dreesen, C.E. de Die‐Smulders, I.F. de Coo. Preventing the transmission of mitochondrial DNA disorders using prenatal or preimplantation genetic diagnosis. *Ann N Y Acad Sci* 2015; 1350:29–36. 10.1111/nyas.1286626312584

21
L. Craven, H.A. Tuppen, G.D. Greggains, S.J. Harbottle, J.L. Murphy, L.M. Cree, et al. Pronuclear transfer in human embryos to prevent transmission of mitochondrial DNA disease. *Nature* 2010; 465(7294): 82–5; M. Tachibana, M. Sparman, H. Sritanaudomchai, H. Ma, L. Clepper, J. Woodward, et al. Mitochondrial gene replacement in primate offspring and embryonic stem cells. *Nature* 2009; 461(7262): 367–72; A. Yabuuchi, Z. Beyhan, N. Kagawa, C. Mori, K. Ezoe, K. Kato, et al. Prevention of mitochondrial disease inheritance by assisted reproductive technologies: Prospects and challenges. *Biochim Biophys Acta BBA ‐ Gen Subj* 2012; 1820(5): 637–42.

22
Here I will not discuss if the ‘mitochondrial donor’ is or is not a mother. For a discussion of such issue see, J.B. Appleby. Should mitochondrial donation be anonymous? *J Med Philos* Forthcoming; R. Brandt. Mitochondrial donation and ‘the right to know.’ *J Med Ethics Published Online First* 2016; 1–8 medethics–2016–103587; M. Piotrowska, M. Why Is An Egg Donor a Genetic Parent, But Not a Mitochondrial Donor? *Camb Q Healthc Ethics* Forthcoming.

23
Nuffield Council on Bioethics. *op. cit* note 17; Tachibana, Sparman, Sritanaudomchai, Ma, Clepper, Woodward, et al. *op. cit* note 21; L.A. Hyslop, P. Blakeley, L. Craven, et al. Towards Clinical Application of Pronuclear Transfer to Prevent Mitochondrial DNA Disease. *Nature* 2016; 534(7607): 383–386; M. Yamada, V. Emmanuele, M.J. Sanchez‐Quintero, et al. Genetic Drift Can Compromise Mitochondrial Replacement by Nuclear Transfer in Human Oocytes’. *Cell Stem Cell* 2016; 18(6): 749–54.

24
Nuffield Council on Bioethics. *op. cit* note 17; Craven, Tuppen, Greggains, Harbottle, Murphy, Cree, et al. *op. cit*. note 21.

25
N. Agar. Liberal Eugenics. *Public Aff Q* 1998; 12(2): 141. 11657329

26
Although in this article I focus on Habermas's account of prenatal genetic therapeutic interventions I will briefly mention his two main arguments (as identified by Jonathan Pugh) *against prenatal genetic enhancing interventions*: the empirical argument and the argument from natality. First, the empirical argument holds that a prenatally genetically enhanced child might feel un‐free to carry on what she has chosen as a life‐project, because the enhancing intervention generates parental expectations regarding which life‐project she must follow. The child might come to believe that since she *incarnates* her parents’ expectations she cannot do otherwise than that that is expected from her. Second, the argument from natality holds that the autonomy of prenatally genetically enhanced children would be undermined, in that the children would not be able to regard themselves as autonomous agents. For Habermas an agent can only regard herself as autonomous if there was a time when she was not subject to the social forces that determine her self, this time is the time before she was born. ‘For a person to be himself, a point of reference is required which goes back beyond the lines of tradition and the contexts of interaction which constitute the process of formation through which personal identity is moulded in the course of a life history.’ Habermas. *op. cit. note 11*, p. 59. Someone could posit that the arguments that Habermas presents against prenatal genetic enhancing interventions could be applied to prenatal genetic therapeutic interventions. This strategy would be unsuccessful if prenatal genetic therapeutic interventions: a) did not impose a parental expectation regarding a specific life‐project unto a child, and b) did not import any value that could affect the prenatal self. Now, it seems that prenatal genetic therapeutic interventions in fact do not impose a parental expectation regarding a specific life‐project; but, on the other hand, it is true that our concept of health is not value‐free, in that it is, at least partly, determined by social values. Habermas could escape this criticism if he acknowledged that in our dealings with prenatal life we cannot escape to always bring with us our conception of what is a good life. If he did this then he would have to renounce to the argument from natality in its present form. See, Pugh. *op. cit*. note 12.

27
Habermas. *op. cit*. note 11, p. 96–97.

28
Ibid: 43.

29
Two caveats should be mentioned in regard to presumed informed consent. First, doctors cannot resort to presumed informed consent to start or carry on treatment when patients have properly manifested their autonomous will not to receive any treatment, or some types of treatments, and healthcare professionals know about this, for example, in the case of ‘do not resuscitate’ orders. Second, the *legal* justificatory reasons for health care professionals to act, on specific circumstances, can, or not, be grounded on the notion of presumed informed consent. For example, in the UK doctors’ legal justification for intervening when patients cannot provide their informed consent *is not* presumed informed consent but rather ‘patients’ best interests’, as established in the *Mental Capacity* Act: Department of Health. 2005. *Mental Capacity Act*. London, HMSO: Sect. 4–5.

30
Habermas. *op. cit*. note 11, p. 63.

31
Ibid: 43.

32
Ibid: 117.

33
Ibid: 69–70.

34
If Habermas were to *reject* all prenatal genetic therapeutic *germline* modifications then what would follow is that the final conclusion of the article would be restricted to cases of selecting for males. Let's remember that mitochondria are only inherited via the maternal line, so choosing for males would amount to a kind of somatic manipulation.

35
An alternative solution to this ‘germline problem’ can be provided by David Boonin's treatment of the ‘Non‐identity Problem’. See D. Boonin. How to Solve the Non‐Identity Problem. *Public Aff Q* 2008; 22(2): 129–59.

36
A central theme in Habermas's *The Future of Human Nature* is the ethical self‐understanding of the species, for which he means that we regard ourselves as creatures that are both grown and made. We are grown insofar as our mental and physical capacities are not determined by other's actions. We are made in that others, and ourselves, can and do exercise control over our lives and capacities. See D. Gunson. What Is the Habermasian Perspective in Bioethics? *Camb Q Healthc Ethics* 2012; 21(2): 190. In this article I will not discuss the relation between the ethical self‐understanding of the species and MRTs. I will not do so because for Habermas the ethical self‐understanding of the species is at stake when *instrumentalization* of the prepersonal life occurs, and this is not the case when we carry on prenatal genetic therapeutic interventions: ‘What is at stake, therefore, with the instrumentalization of prepersonal life [by means of genetic engineering] is the ethical self‐understanding of the species, which is crucial for whether we may go on to see ourselves as beings committed to moral judgement and action.’ Habermas. *op. cit*. note 11, p. 71. It must be said that even when PNT uses human embryos for the sake of other human embryos, this is not the kind of instrumentalization that Habermas is exploring in the previous quote.

37
S. Wilkinson. 2010. *Choosing Tomorrow's Children: The Ethics of Selective Reproduction*. Oxford: Oxford University Press; 191.

38
Nuffield Council on Bioethics. *op. cit*. note 17. p. vii.

39
S.C. Sallevelt, J.C. Dreesen, M. Drüsedau, S. Spierts, E. Coonen, F.H. van Tienen, et al. Preimplantation genetic diagnosis in mitochondrial DNA disorders: challenge and success. *J Med Genet* 2013; 50(2): 125–32. 10.1136/jmedgenet-2012-10117223339111

40
Sallevelt, Dreesen, Drüsedau, Spierts, Coonen, van Tienen, et al. *op. cit* note 39; A.L. Bredenoord, W. Dondorp, G. Pennings, C. de Die‐Smulders, B. Smeets, G. de Wert. Preimplantation genetic diagnosis for mitochondrial DNA disorders: ethical guidance for clinical practice. *Eur J Hum Genet* 2009; 17(12): 1550–9. 10.1038/ejhg.2009.88PMC298702419471315

41
T. Pal, J. Permuth‐Wey, J.A. Betts, J.P. Krischer, J. Fiorica, H. Arango, et al. BRCA1 and BRCA2 mutations account for a large proportion of ovarian carcinoma cases. *Cancer* 2005; 104(12): 2807–16. 10.1002/cncr.2153616284991

42
Habermas. *op. cit*. note 11, p. 43,63, 91.

43
Ibid: 21.

44
Ibid: 12, 51, 52.

45
According to Elizabeth Barnes the ‘bad‐difference view’ maintains ‘that disability is by itself something that makes you worse off (…) [while for the] mere‐difference views of disability, having a disability makes you nonstandard or different, but it doesn't by itself make you worse off’. E. Barnes. Valuing Disability, Causing Disability. *Ethics* 2014; 125(1): 89.

46
R. Cooper. Can It Be a Good Thing to Be Deaf? *J Med Philos* 2007; 32(6): 563–83. 10.1080/0360531070168094018027249

47
Habermas. *op. cit*. note 11, p. 63.

48
Ibid: 52.

49
Ibid: 34–35.

50
Ibid: 43.

51
Ibid: 31.

52
Here we must note a hermeneutical complexity, this is: what does ‘not to be disposed over’ mean? In the English translation of *The Future of Human Nature* it is not completely clear what should we make of such a phrase. The German word ‘unverfügbar’ can, alternatively, be translated as ‘unavailable’ but even if we do so its meaning remains unclear. In this context ‘unverfügbar’ seems to better translate as ‘not to use/not to access’.

53
It is true that someone could reject Habermas's account of the value of human prepersonal life, endorse a liberal stance regarding the destruction of human embryos, and hold a Habermasian stance in regards to prenatal genetic interventions.

